# Hypertensive disorders of pregnant women with heart disease: the ESC EORP ROPAC Registry^[Author-notes ehac308-FM2]^

**DOI:** 10.1093/eurheartj/ehac308

**Published:** 2022-06-21

**Authors:** Karishma P Ramlakhan, Isabelle Malhamé, Ariane Marelli, Tobias Rutz, Sorel Goland, Arie Franx, Karen Sliwa, Uri Elkayam, Mark R Johnson, Roger Hall, Jérôme Cornette, Jolien W Roos-Hesselink

**Affiliations:** Department of Cardiology, Erasmus MC, University Medical Center Rotterdam, Rg-435 - P.O. Box: 2040, Rotterdam, 3000 CA, The Netherlands; Department of Obstetrics and Fetal Medicine, Erasmus MC—Sophia’s Children’s Hospital, University Medical Center Rotterdam, Rotterdam, 3000 CB, The Netherlands; Department of Medicine, McGill University Health Centre, Montreal, QC H4A 3J1, Canada; McGill Adult Unit for Congenital Heart Disease (MAUDE Unit), Department of Medicine, McGill University Health Centre, Montreal, QC H4A 3J1, Canada; Service of Cardiology, Lausanne University Hospital and University of Lausanne, Lausanne, CH-1011, Switzerland; Heart Institute, Kaplan Medical Center, Rehovot, Hebrew University and Hadassah Medical School, Rehovot, 76100 and Jerusalem, 9112102, Israel; Department of Obstetrics and Fetal Medicine, Erasmus MC—Sophia’s Children’s Hospital, University Medical Center Rotterdam, Rotterdam, 3000 CB, The Netherlands; Cape Heart Institute, Department of Medicine and Cardiology, University of Cape Town, Cape Town, 7925, South Africa; Department of Medicine, Division of Cardiovascular Medicine and Department of Obstetrics and Gynecology, University of Southern California, Keck School of Medicine, Los Angeles, CA 90033, United States; Department of Obstetric Medicine, Imperial College London, Chelsea and Westminster Hospital, London SW7 2BX, United Kingdom; Department of Cardiology, Norwich Medical School, University of East Anglia, Norwich NR4 7TJ, United Kingdom; Department of Obstetrics and Fetal Medicine, Erasmus MC—Sophia’s Children’s Hospital, University Medical Center Rotterdam, Rotterdam, 3000 CB, The Netherlands; Department of Cardiology, Erasmus MC, University Medical Center Rotterdam, Rg-435 - P.O. Box: 2040, Rotterdam, 3000 CA, The Netherlands

**Keywords:** Pregnancy, Hypertensive disorders of pregnancy, Pre-eclampsia, Maternal mortality, Maternal morbidity

## Abstract

**Aims:**

Hypertensive disorders of pregnancy (HDP) occur in 10% of pregnancies in the general population, pre-eclampsia specifically in 3–5%. Hypertensive disorders of pregnancy may have a high prevalence in, and be poorly tolerated by, women with heart disease.

**Methods and results:**

The prevalence and outcomes of HDP (chronic hypertension, gestational hypertension or pre-eclampsia) were assessed in the ESC EORP ROPAC (*n* = 5739), a worldwide prospective registry of pregnancies in women with heart disease.

The overall prevalence of HDP was 10.3%, made up of chronic hypertension (5.9%), gestational hypertension (1.3%), and pre-eclampsia (3%), with significant differences between the types of underlying heart disease (*P* < 0.05). Pre-eclampsia rates were highest in women with pulmonary arterial hypertension (PAH) (11.1%), cardiomyopathy (CMP) (7.1%), and ischaemic heart disease (IHD) (6.3%). Maternal mortality was 1.4 and 0.6% in women with vs. without HDP (*P* = 0.04), and even 3.5% in those with pre-eclampsia. All pre-eclampsia-related deaths were post-partum and 50% were due to heart failure. Heart failure occurred in 18.5 vs. 10.6% of women with vs. without HDP (*P* < 0.001) and in 29.1% of those with pre-eclampsia. Perinatal mortality was 3.1 vs. 1.7% in women with vs. without HDP (*P* = 0.019) and 4.7% in those with pre-eclampsia.

**Conclusion:**

Hypertensive disorders of pregnancy and pre-eclampsia rates were higher in women with CMP, IHD, and PAH than in the general population. Adverse outcomes were increased in women with HDP, and maternal mortality was strikingly high in women with pre-eclampsia. The combination of HDP and heart disease should prompt close surveillance in a multidisciplinary context and the diagnosis of pre-eclampsia requires hospital admission and continued monitoring during the post-partum period.

## Structured Graphical Abstract

**Structured graphical abstract:**
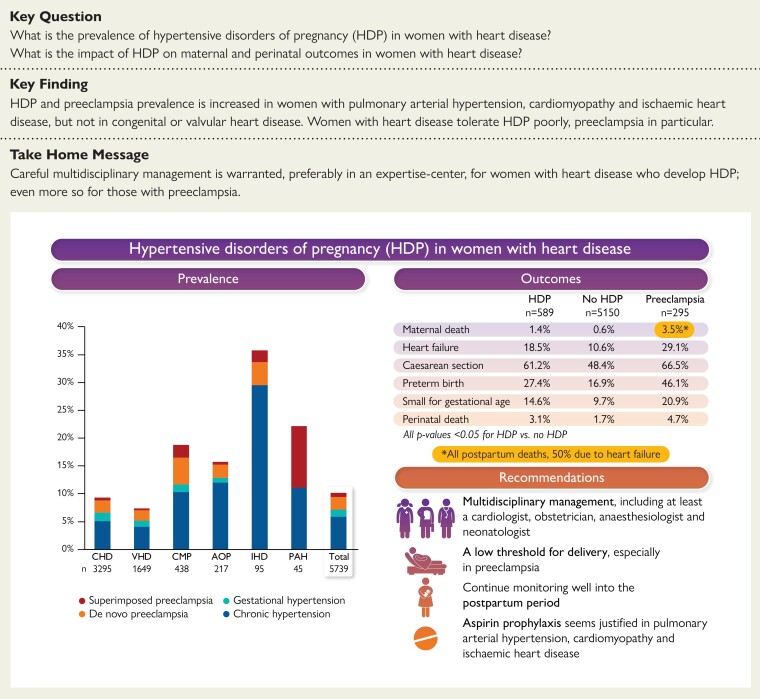
Hypertensive disorders of pregnancy (HDP) in women with heart disease.

**See the editorial comment for this article ‘Understanding the implications of hypertensive disorders in pregnancy in women with heart disease’, by Matthew Cauldwell, https://doi.org/10.1093/eurheartj/ehac406.**

## Introduction

Hypertensive disorders of pregnancy (HDP) include pre-existing hypertension (chronic hypertension) and de novo hypertension during pregnancy (gestational hypertension and pre-eclampsia). Hypertensive disorders of pregnancy affect up to 10% of pregnancies globally^1,2^ and can induce severe dysfunction in every component of the cardiovascular system. Women with structural heart disease, particularly ischaemic heart disease (IHD) or certain types of congenital heart disease (CHD), are more likely to have chronic hypertension.^3^ Moreover, they may be at higher risk of developing HDP, given shared risk factors between heart disease and HDP. These shared risk predispositions include maternal age ≥35, obesity, and diabetes.^2–4^ In addition, cardiac dysfunction might be a primary trigger in the aetiology of pre-eclampsia,^5^ although the exact mechanism hereof remains unknown.

Heart disease and HDP are both major causes of severe maternal morbidity,^6^ mortality,^7,8^ and perinatal complications.^7,9^ In combination, they are likely to produce an even worse pregnancy outcome. Indeed, pre-eclampsia increases the risk of heart failure in its own right,^10^ and it seems very likely that the clinical manifestations of pre-eclampsia such as capillary leak, coagulation disorders, fluctuations in blood pressure, and systolic and diastolic dysfunctions, would be poorly tolerated in women with heart disease.^11^

Existing data on the prevalence and outcome of HDP in women with heart disease is confined mostly to retrospective studies with heterogeneity in the definition of HDP, with chronic hypertension variably included as a baseline characteristic, or as an outcome.^12–14^ The few prospective studies are limited by small sample size.^15,16^ The Registry of Pregnancy and Cardiac disease (ROPAC) is currently the largest prospective international registry of pregnancies in women with structural heart disease.^3^ In this study, we sought to establish the prevalence of HDP and their impact on pregnancy outcome in women with various types of structural heart disease. In addition, we aimed to elucidate possible interregional differences.

## Methods

The ROPAC was initiated in 2007 by the European Society of Cardiology (ESC) as part of the EURObservational Research Programme.^3^ From January 2007 to January 2018, a total of 5739 pregnancies were included from 53 countries.

### Data source and study definitions

A detailed description of the study protocol and design has been published previously.^17^ Participating centres managed the approvals of national or regional ethics committees or Institutional Review Boards, according to local regulations. The ROPAC prospectively included pregnant women with structural heart disease, divided into six diagnostic groups: CHD, valvular heart disease (VHD), or IHD, cardiomyopathy (CMP), aortopathy (AOP), and pulmonary arterial hypertension (PAH). Non-structural heart diseases such as arrhythmia were not included. Pulmonary arterial hypertension was the main diagnosis in the case of primary PAH, not when secondary to e.g. CHD or lung disease. Baseline characteristics before pregnancy included age, parity, country, primary cardiac diagnosis and concomitant disease, prior interventions and cardiovascular risk factors, including chronic hypertension, diabetes, and smoking, New York Heart Association (NYHA) functional classification and cardiac medication. Maternal cardiovascular risk was classified according to the modified World Health Organization classification scale.^18^ Countries were grouped into geographic regions as in the Report and Statistical Annex of the Sustainable Development Goals, and as low- or middle-income countries (LMICs) or high-income countries (HICs) according to their economic status in The International Monetary Fund classification.^19^

Outcomes were assessed by the local investigator for each participating centre, using patient record files and clinical information, and examined during pregnancy until 6 months post-partum. All outcomes were as defined in the ROPAC from its initiation in 2007, except for HDP, which were redefined to conform to the 2018 International Society for the Study of Hypertension in Pregnancy (ISSHP) definitions during the analyses.^2^ Hypertension was defined as systolic blood pressure ≥140 mmHg and/or diastolic blood pressure ≥90 mmHg.^2,18,20^ Chronic hypertension was defined as hypertension pre-dating pregnancy or diagnosed before 20 weeks of gestation. Gestational hypertension was defined as *de novo* hypertension occurring after 20 weeks of gestation in the absence of features of pre-eclampsia. Pre-eclampsia was defined as the presence of de novo hypertension after 20 weeks’ gestation accompanied by proteinuria and/or evidence of maternal acute kidney injury, liver dysfunction, neurological features, haemolysis or thrombocytopaenia, or foetal growth restriction. Haemolysis, elevated liver enzymes and low platelets (HELLP) syndrome and eclampsia (the occurrence of convulsions) were considered as severe manifestations of pre-eclampsia and were not classified individually. Superimposed pre-eclampsia was defined as chronic hypertension complicated by maternal organ dysfunction consistent with pre-eclampsia. Hypertensive disorders of pregnancy were classified hierarchically, based on HDP status at the end of pregnancy, without overlap: a pregnancy with pre-eclampsia following chronic hypertension was classified as (superimposed) pre-eclampsia and not as chronic hypertension, and pre-eclampsia following gestational hypertension was considered pre-eclampsia and not gestational hypertension. This classification differs from the earlier ROPAC publications to conform to the current ISSHP guidelines.^2,3^ Early-onset pre-eclampsia was defined as pre-eclampsia occurring at <34 weeks of gestation, emergency Caesarean section as being performed <24 h after the decision to deliver, and maternal mortality as death during pregnancy or <6 months after delivery. Late foetal mortality was defined as foetal mortality >24 weeks, preterm birth as delivery <37 weeks, low Apgar as a score <7 at 5 min, small for gestational age as birth weight <10th centile according to regional normal values and perinatal mortality as late foetal mortality and neonatal mortality combined.

### Statistical analysis

Baseline characteristics and the prevalence of HDP were described for the whole population and compared between the six diagnostic groups, as well as between geographic regions and economic status. Maternal and perinatal outcomes were compared between women with and without HDP, between geographic regions and economic status. Baseline characteristics were compared between early- and late-onset pre-eclampsia. Categorical data are presented as percentages of the total number of pregnancies. Differences between groups were compared using χ^2^ tests. Continuous data are presented as mean and standard deviation if normally distributed, or as median and interquartile range (Q1–Q3) if skewed, which was examined with Q–Q plots. Differences between two groups were measured using independent samples *t*-tests or Mann–Whitney *U* tests, as appropriate. Differences between multiple groups were assessed with one-way ANOVA tests.

We evaluated associations between baseline characteristics with pre-eclampsia (including de novo and superimposed pre-eclampsia) in women with heart disease using univariate and multivariable logistic regression. Variables that were entered into the multivariable analysis were those that had a *P*-value <0.1 in the univariable logistic regression analysis or that were known to be associated with pre-eclampsia regardless of *P*-value (multiple gestation and smoking were forced into the model). Owing to multicollinearity between the baseline variables and the diagnostic groups, we performed a separate univariable regression analysis to test the difference of diagnostic groups, compared with CHD, on pre-eclampsia. Associations are presented as odds ratios (ORs) with 95% confidence intervals (CIs) and *P*-value. Missing data were missing at random and handled with multiple imputation for the following variables: age, body mass index, nulliparity, smoking, chronic hypertension, diabetes mellitus, signs of heart failure, HDP in a previous pregnancy, and gestational diabetes mellitus. A two-sided *P*-value <0.05 was considered significant for all analyses. All analyses were performed using IBM SPSS Statistics version 25.0 (IBM Corp).

## Results

The total ROPAC cohort of 5739 pregnancies (mean age 29.5 years) was divided into six diagnostic groups, for which baseline characteristics are displayed in Supplementary material online, *Table S1*: CHD (*n* = 3295, 57.4%), VHD (*n* = 1649, 28.7%), CMP (*n* = 438, 7.6%), AOP (*n* = 217, 3.8%), IHD (*n* = 95, 1.7%), and PAH (*n* = 45, 0.8%).^3^ Cardiac medication that was used before and during pregnancy in women with HDP is listed in Supplementary material online, *Table S2*, which involved beta blockers in 26.3% and calcium channel blockers in 9.5% during pregnancy.

**Figure 1:**
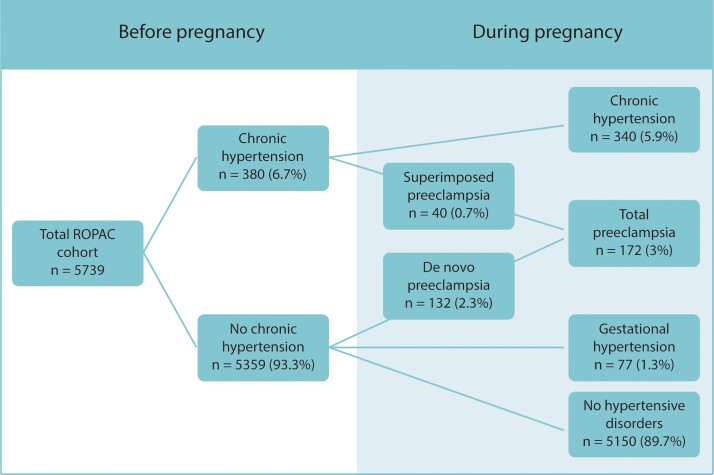
Hypertensive disorders of pregnancy in women with structural heart disease. ROPAC, Registry of Pregnancy and Cardiac disease.

**Figure 2:**
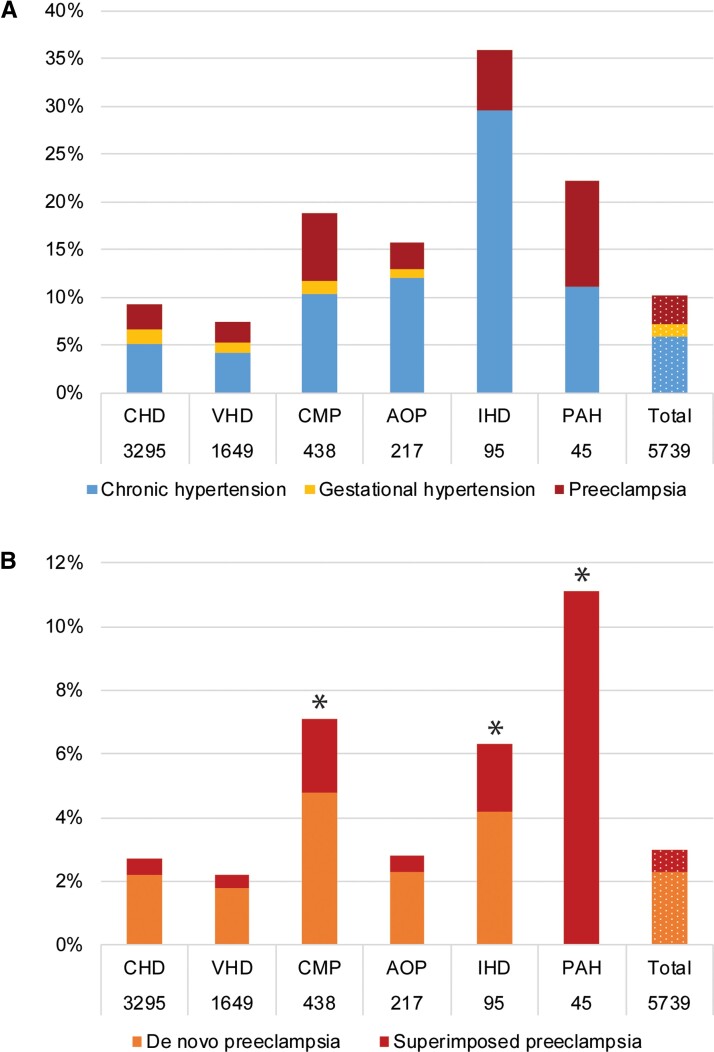
Hypertensive disorders of pregnancy per diagnostic group. * denotes the diagnostics groups with significantly increased risk of pre-eclampsia, with CHD as reference. AOP, aortic pathology; CHD, congenital heart disease; CMP, cardiomyopathy; IHD, ischaemic heart disease; PAH, pulmonary arterial hypertension; VHD, valvular heart disease.

The overall prevalence of chronic hypertension was 5.9%, the prevalence of gestational hypertension was 1.3%, superimposed pre-eclampsia was 0.7%, and de novo pre-eclampsia 2.3%. Thus, the total prevalence of HDP in women with structural heart disease was 10.3% (*Figure 1*). *Table 1* and *Figure 2* show HDP prevalence for the specific diagnostic groups. The HDP prevalence was 9.3% in CHD, 7.5% in VHD, 18.7% in CMP, 15.7% in AOP, 35.8% in IHD, and 22.2% in PAH (*Figure 2A*). The prevalence of pre-eclampsia was 2.6% in CHD, 2.2% in VHD, 7.1% in CMP, 2.8% for AOP, 6.3% in IHD, and 11.1% in PAH (*Figure 2B*).

**Table 1:** Main diagnosis of structural heart disease and the prevalence of hypertensive disorders of pregnancy

	n	Chronic hypertension	Gestational hypertension	Pre-eclampsia*	Total HDP
**Total cohort**	**5739**	**340** (**5.9)**	**77** (**1.3)**	**172** (**3)**	**589** (**10.3)**
**CHD**	**3295**	**168** (**5.1)**	**51** (**1.5)**	**87** (**2.6)**	**306** (**9.3)**
Eisenmenger	31	1 (3.2)	0 (0)	1 (3.2)	2 (6.5)
Fontan circulation	54	1 (1.9)	0 (0)	1 (1.9)	2 (3.7)
Pulmonary atresia	32	1 (3.1)	1 (3.1)	5 (15.6)	7 (21.9)
Double outlet right ventricle	27	1 (3.7)	0 (0)	0 (0)	1 (3.7)
Tetralogy of Fallot	426	8 (1.9)	7 (1.6)	6 (1.4)	21 (4.9)
Congenitally corrected TGA	39	1 (2.6)	2 (5.1)	0 (0)	3 (7.7)
Atrial switch for TGA	121	1 (0.8)	2 (1.7)	4 (3.3)	7 (5.8)
Arterial switch for TGA	41	1 (2.4)	0 (0)	2 (4.9)	3 (7.3)
Aortic coarctation	303	75 (24.8)	8 (2.6)	8 (2.6)	91 (30)
Atrioventricular septal defect	169	1 (0.6)	1 (0.6)	3 (1.8)	5 (3)
Atrial septal defect	495	18 (3.6)	7 (1.4)	15 (3)	40 (8.1)
Ventricular septal defect	463	13 (2.8)	7 (1.5)	12 (2.6)	32 (6.9)
Ebstein’s anomaly	80	7 (8.8)	1 (1.3)	0 (0)	8 (10
Aortic valve abnormality	267	14 (5.2)	5 (1.9)	11 (4.1)	30 (11.2)
Pulmonary valve abnormality	206	4 (1.9)	4 (1.9)	5 (2.4)	13 (6.3)
Mitral valve abnormality	83	3 (3.6)	1 (1.2)	0 (0)	4 (4.8)
Pulmonary vein abnormality	33	0 (0)	0 (0)	1 (3)	1 (3)
Patent ductus arteriosus	71	4 (5.6)	1 (1.4)	1 (1.4)	6 (8.5)
Other/unknown CHD	354	14 (4)	4 (1.1)	12 (3.4)	30 (8.5)
**VHD**	**1649**	**68** (**4.1)**	**18** (**1.1)**	**37** (**2.2)**	**123** (**7.5)**
Aortic stenosis	138	10 (7.2)	3 (2.2)	7 (5.1)	20 (14.5)
Aortic regurgitation	148	12 (8.1)	5 (3.4)	5 (3.4)	22 (14.9)
Mixed aortic disease	70	5 (7.1)	0 (0)	2 (2.9)	7 (10)
Mitral stenosis	288	11 (3.8)	0 (0)	3 (1)	14 (4.9)
Mitral regurgitation	500	22 (4.4)	7 (1.4)	7 (1.4)	36 (7.2)
Mixed mitral disease	278	4 (1.4)	1 (0.4)	7 (2.5)	12 (4.3)
Pulmonary stenosis	102	2 (2)	1 (1)	3 (2.9)	6 (5.9)
Pulmonary regurgitation	10	1 (10)	1 (10)	0 (0)	2 (20)
Other/unknown VHD	115	1 (0.9)	0 (0)	3 (2.6)	4 (3.5)
**CMP**	**438**	**45** (**10.3)**	**6** (**1.4)**	**31** (**7.1)**	**82** (**18.7)**
Dilated CMP	84	13 (15.5)	1 (1.2)	6 (7.1)	20 (23.8)
Hypertrophic CMP	93	7 (7.5)	1 (1.1)	1 (1.1)	9 (9.7)
Peripartum CMP	59	7 (11.9)	2 (3.4)	6 (10.2)	15 (25.4)
Myocarditis	19	1 (5.3)	2 (10.5)	1 (5.3)	4 (21.1)
Other/unknown CMP	183	17 (9.3)	0 (0)	17 (9.3)	34 (18.6)
*AOP*	**217**	**26** (**12)**	**2** (**0.9)**	**6** (**2.8)**	**34** (**15.7)**
Marfan syndrome	100	12 (12)	2 (2)	2 (2)	16 (16)
EDS Type IV	4	1 (25)	0 (0)	0 (0)	1 (25)
Bicuspid aortic valve	44	2 (4.5)	0 (0)	1 (2.3)	3 (6.8)
Turner syndrome	16	4 (25)	0 (0)	0 (0)	4 (25)
FTAAD	3	1 (33.3)	0 (0)	0 (0)	1 (33.3)
Other/unknown AOP	50	6 (12)	1 (2)	3 (6)	10 (20.4)
**IHD**	**95**	**28** (**29.5)**	**0** (**0)**	**6** (**6.3)**	**34** (**35.8)**
**PAH**	**45**	**5** (**11.1)**	**0** (**0)**	**5** (**11.1)**	**10** (**22.2)**
Idiopathic/heritable	25	4 (16)	0 (0)	2 (8)	6 (24)
Connective tissue disease	3	0 (0)	0 (0)	0 (0)	0 (0)
Chronic thromboembolic	4	0 (0)	0 (0)	0 (0)	0 (0)
Lung disease	2	1 (50)	0 (0)	0 (0)	1 (50)
Other/unknown PAH	11	0 (0)	0 (0)	3 (27.3)	3 (27.3)
P-value between diagnostic groups*		<0.001	0.545	<0.001	<0.001

Data are *n* (%). *P*-values were calculated using χ^2^ tests for the comparison between CHD, VHD, CMP, AOP, IHD, and PAH, significant if at least one of the groups is significantly different compared with the other groups. * Pre-eclampsia includes superimposed and de novo pre-eclampsia. AOP, aortic pathology; CHD, congenital heart disease; CMP, cardiomyopathy; EDS, Ehlers-Danlos Syndrome; FTAAD, Familial Thoracic Aortic Aneurysm and Dissection; HDP, hypertensive disorders of pregnancy (defined as gestational hypertension and pre-eclampsia); IHD, ischaemic heart disease; PAH, pulmonary arterial hypertension; TGA, transposition of the great arteries; VHD, valvular heart disease.

The highest prevalence of pre-eclampsia (>5%) was observed in pulmonary atresia (15.6%), PAH (11.1%), peripartum CMP (10.2%), dilated CMP (7.1%), IHD (6.3%), myocarditis (5.3%), and aortic stenosis (5.1%) (*Table 1*). Despite the prevalence of chronic hypertension at baseline ranging from 4.6% (VHD) to 31.5% (IHD) between the groups (see Supplementary material online, *Table S1*), superimposed pre-eclampsia was <2.5% in all groups except in PAH, where 50% of pre-eclampsia was superimposed pre-eclampsia (11.1%) (*Figure 2B* and Supplementary material online, *Table S3*).

The gestational age at which pre-eclampsia was diagnosed was known in 86 of 172 pregnancies, of which 26% were early-onset and 74% late-onset (*Table 2*). Women with early-onset pre-eclampsia more often had chronic hypertension (54.5 vs. 21.9%, *P* = 0.004), LV dysfunction (13.6 vs. 1.6%, *P* = 0.02), and NYHA Class > II (18.2 vs. 3.1%, *P* = 0.017) compared with women who developed late pre-eclampsia.

**Table 2:** Comparison of baseline characteristics between early and late pre-eclampsia

	Early PE *n* = 22 (26%)	Late PE *n* = 64 (74%)	*P*-value
Age, years (Q1–Q3)	35.4 (25.6–41.2)	28.9 (24.4–35.3)	0.075
BMI, kg/m^2^(Q1–Q3)	26.9 (23.2–31.6)	25 (22.3–29.1)	0.447
Nulliparity	10 (45.5)	39 (60.9)	0.206
Multiple pregnancy	1 (4.5)	1 (1.6)	0.423
LMIC	13 (59.1)	27 (42.2)	0.170
Current smoker	2 (10.5)	4 (7)	0.623
Chronic hypertension	12 (54.5)	14 (21.9)	**0**.**004**
Diabetes mellitus Type 1/2	0 (0)	3 (4.8)	0.293
Atrial fibrillation	0 (0)	0 (0)	n.a.
Signs of heart failure	6 (28.6)	8 (12.5)	0.085
Estimated LVEF <40%	3 (13.6)	1 (1.6)	**0**.**020**
NYHA Class >II	4 (18.2)	2 (3.1)	**0**.**017**
mWHO Class >II	18 (81.8)	46 (71.9)	0.356
Cardiac medication use	17 (77.3)	38 (59.4)	0.131
Prior cardiac intervention	8 (36.4)	35 (55.6)	0.121
HDP in previous pregnancy	3 (14.3)	7 (11.3)	0.716
Aspirin use in current pregnancy	1 (4.5)	10 (15.6)	0.180
GDM in current pregnancy	2 (9.1)	7 (10.9)	0.807
CHD	5 (22.7)	38 (59.4)	**0**.**003**
VHD	7 (31.8)	8 (12.5)	**0**.**039**
CMP	7 (31.8)	8 (12.5)	**0**.**039**
AOP	1 (4.5)	5 (7.8)	0.604
IHD	0 (0)	3 (4.7)	0.301
PAH	2 (9.1)	2 (3.1)	0.252

Data are *n* (%), unless otherwise specified. Bold denotes *P* < 0.05. *P*-values calculated between women with early- and late-onset pre-eclampsia, using χ^2^ tests, unpaired *t*-tests and Mann–Whitney *U* tests as appropriate. AOP, aortic pathology; BMI, body mass index; CHD, congenital heart disease; CMP, cardiomyopathy; GDM, gestational diabetes mellitus; HDP, hypertensive disorders of pregnancy (defined as gestational hypertension and pre-eclampsia); IHD, ischaemic heart disease; LMIC, low-/middle-income country; LVEF, left ventricular ejection fraction; mWHO, modified World Health Organization classification for maternal cardiovascular risk; NYHA, New York Heart Association functional classification; PAH, pulmonary arterial hypertension; VHD, valvular heart disease.

**Figure 3:**
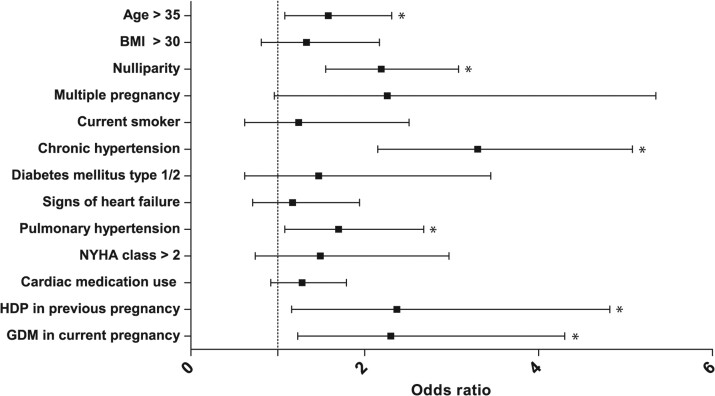
Multivariable regression analysis of predictors of pre-eclampsia in women with structural heart disease. *P < 0.05. BMI, body mass index; GDM, gestational diabetes mellitus; HDP, hypertensive disorders of pregnancy; NYHA, New York Heart Association.

The results of the univariable and multivariable regression analyses evaluating baseline characteristics’ associations with pre-eclampsia (including both superimposed and de novo) are listed in Supplementary material online, *Table S4*. Chronic hypertension (OR: 3.3, 95% CI: 2.2–5.1), HDP in a previous pregnancy (OR: 2.4, 95% CI: 1.2–4.8), gestational diabetes in the current pregnancy (OR: 2.3, 95% CI: 1.2–4.3), nulliparity (OR: 2.2, 95% CI: 1.6–3.1), pulmonary hypertension (OR: 1.7, 95% CI: 1.1–2.7), and maternal age >35 (OR: 1.6, 95% CI: 1.1–2.3) were significantly associated with pre-eclampsia (*Figure 3*). In the separate univariable analysis on diagnostic groups and pre-eclampsia prevalence, PAH (OR: 4.6, 95% CI: 1.8–12), CMP (OR: 2.8, 95% CI: 1.8–4.3), and IHD (OR: 2.5, 95% CI: 1.1–5.8) were higher compared with CHD.

Maternal and perinatal outcomes for all types of HDP are described in *Table 3*. In women with HDP, compared with those without, maternal mortality occurred more often (1.4 vs. 0.6%, *P* = 0.042), as did heart failure (18.5 vs. 10.6%, *P* < 0.001), Caesarean section (61.2 vs. 48.4%, *P* < 0.001), preterm birth (27.4 vs. 16.9%, *P* < 0.001), low Apgar score (9.8 vs. 6.6%, *P* < 0.001), small for gestational age (14.6 vs. 9.7%, *P* < 0.001) and perinatal mortality (3.1 vs. 1.7%, *P* = 0.019). In women with pre-eclampsia and heart disease, maternal mortality was (3.5 vs. 0.6% in those without pre-eclampsia, *P* < 0.001) and heart failure occurred in (29.1 vs. 10.9%, *P* < 0.001). The six cases of maternal death in women with pre-eclampsia all occurred post-partum and were due to heart failure (*n* = 3), pulmonary embolism (*n* = 1), pneumonia (*n* = 1), and unknown causes (*n* = 1) (*Table 4*).

**Table 3:** Maternal and perinatal outcomes

	No HDP (*n* = 5150)	Any HDP (*n* = 589)	*P*-value	Chronic hypertension (*n* = 340)	Gestational hypertension (*n* = 77)	Pre-eclampsia (*n* = 172)
**Maternal outcomes**						
Maternal mortality	32 (0.6)	8 (1.4)	**0**.**042**	2 (0.6)	0 (0)	6 (3.5)
Heart failure	546 (10.6)	109 (18.5)	**<0**.**001**	47 (13.8)	12 (15.6)	50 (29.1)
Caesarean section	2336 (48.4)	345 (61.2)	**<0**.**001**	199 (62.6)	33 (43.4)	113 (66.5)
Emergency CS	657 (12.8)	109 (18.5)	**<0**.**001**	49 (14.4)	7 (9.1)	53 (30.8)
Emergency CS for cardiac reasons	111 (2.2)	21 (3.6)	**0**.**031**	10 (2.9)	0 (0)	11 (6.4)
Post-partum haemorrhage	152 (3)	18 (3.1)	0.887	8 (2.4)	2 (2.6)	8 (4.7)
**Perinatal outcomes**						
Late foetal mortality	64 (1.2)	8 (1.4)	0.730	4 (1.2)	0 (0)	4 (2.3)
Preterm delivery	759 (16.9)	146 (27.4)	**<0**.**001**	61 (20.5)	8 (11.6)	77 (46.1)
Apgar score <7	339 (6.6)	58 (9.8)	**0**.**003**	24 (7.1)	5 (6.5)	29 (16.9)
Small for gestational age	498 (9.7)	86 (14.6)	**<0**.**001**	50 (14.7)	0 (0)	36 (20.9)
Foetal congenital heart disease	143 (2.8)	13 (2.2)	0.421	6 (1.8)	4 (5.2)	3 (1.7)
Neonatal mortality	23 (0.4)	10 (1.7)	**<0**.**001**	6 (1.8)	0 (0)	4 (2.3)
Total perinatal mortality	87 (1.7)	18 (3.1)	**0**.**019**	10 (2.9)	0 (0)	8 (4.7)

Data are *n* (%), bold denotes *P* < 0.05. *P*-values were calculated between HDP and no HDP using χ^2^ tests. CS, Caesarean section; HDP, hypertensive disorders of pregnancy.

**Table 4:** Maternal deaths in women with heart disease and pre-eclampsia

No.	Diagnosis, comorbidities, and medication	Pre-pregnancy cardiac function	Maternal complications	Perinatal complications
1	Dilated CMP, PAH secondary to VSD, severe mitral regurgitation, morbid obesity	NYHA IV LVEF <40% Signs of HF: Yes	G1P0. Onset PE at 34 + 6 weeks, heart failure at 36 + 1, treated with carvedilol and furosemide. 37 + 5 emergency CS. During delivery complaints of severe dyspnoea and haemoptysis, for which intubation and transfer to ICU. In ICU pulmonary oedema and LVEF 25%. Death +2 weeks post-partum due to heart failure and respiratory failure.	SGA
2	Dilated CMP, PAH, non-Hodgkin lymphoma (in remission)	NYHA I LVEF >40% Signs of HF: No	G1P0. Onset PE unknown. Spontaneous vaginal delivery at 40 + 3 weeks Death +3 weeks post-partum, cause unknown.	Neonatal death at + 3 weeks post-partum, cause unknown.
3	Rheumatic VHD (mechanoprosthesis in mitral position with stenosis and regurgitation, aortic regurgitation), PAH **Medication:** Pre-pregnancy use of warfarin was switched to LMWH in the first trimester	NYHA IV LVEF >40% Signs of HF: Yes	G3P1. Onset PE unknown. Spontaneous preterm vaginal birth at 29 + 6 weeks, stillbirth. Complaints of hypertension, dyspnoea, haemoptysis in context of right-sided heart failure with PAH, treated with tadalafil, bosentan and dopamine Death +0 days post-partum	Preterm, intra-uterine foetal demise
4	VHD (tricuspid regurgitation and pulmonary regurgitation), PAH, chronic hypertension	NYHA III LVEF >40% signs HF: Yes	G2P0. Onset PE at 29 weeks, heart failure at 29 + 1, eCS at 29 + 2 Death +1 week post-partum due to right-sided heart failure	Preterm, SGA, low Apgar (3 at 1 min), perinatal death
5	PAH, obesity, chronic hypertension	NYHA IV LVEF >40% Signs of HF: No	G1P0. HELLP syndrome and pulmonary embolism at 36 + 6 weeks, CS at 37 weeks Death +1 day post-partum due to massive pulmonary embolism.	None
6	CHD (Eisenmenger syndrome), maternal age (39 years) **Medication:** Sildenafil	NYHA I LVEF >40% signs HF: No	G1P0. HELLP syndrome at 26 + 6 weeks, CS at 30 weeks. Death +7 weeks post-partum due to respiratory failure after hospital-acquired pneumonia.	Preterm and SGA

CHD, congenital heart disease; CS, Caesarean section; CMP, cardiomyopathy; HELLP syndrome, haemolysis, elevated liver enzymes and low platelets (HELLP) syndrome; HF, heart failure; LVEF, left ventricular ejection fraction; NYHA, New York Heart Association functional classification; PAH, pulmonary arterial hypertension; SGA, small for gestational age; VHD, valvular heart disease.

For the pregnancies in which the gestational age at the diagnosis of pre-eclampsia was known (86 of 172), the outcomes of women with early vs. late pre-eclampsia are presented in Supplementary material online, *Table S5*. Women with early pre-eclampsia had more Caesarean sections (85 vs. 57.8%, *P* = 0.027) and preterm births (70 vs. 29.7%, *P* = 0.001) than women with late pre-eclampsia. The comparison between economic classification and geographic regions for the prevalence of HDP, and the outcomes of women with HDP, is presented in *Table 5* and Supplementary material online, *Table S6* and Supplementary material online, *Figure S1*. There was more superimposed pre-eclampsia, heart failure and late foetal mortality and less foetal CHD observed in LMIC compared with HIC (*Table 5*). There was a significant difference in the prevalence of all HDP types between geographic regions (see Supplementary material online, *Figure S1*). Maternal mortality was highest in Eastern and Southeasthern Asia, and heart failure was highest in Southern Africa (see Supplementary material online, *Table S6*).

**Table 5:** Prevalence and outcome of HDP stratified by low-, middle-, and high-income countries

	LMIC *n* = 2281	HIC *n* = 3458	*P*-value
**Prevalence**			
Chronic hypertension	144 (6.3)	196 (5.7)	0.311
Gestational hypertension	29 (1.3)	48 (1.4)	0.707
Total pre-eclampsia	72 (3.2)	100 (2.9)	0.565
De novo pre-eclampsia	49 (2.1)	83 (2.4)	0.533
Superimposed pre-eclampsia	23 (1)	17 (0.5)	**0**.**021**
Total HDP	245 (10.7)	344 (9.9)	0.333
**Outcomes in women with HDP**	***n* = 245**	***n* = 344**	***P*-value**
**Maternal outcomes**			
Maternal mortality	5 (2)	3 (0.9)	0.227
Heart failure	75 (30.6)	34 (9.9)	**<0**.**001**
Caesarean section	153 (65.9)	192 (57.8)	0.052
Emergency CS	47 (19.2)	62 (18)	0.721
Emergency CS for cardiac reasons	12 (4.9)	9 (2.6)	0.141
Post-partum haemorrhage	7 (2.9)	11 (3.2)	0.813
**Perinatal outcomes**			
Late foetal mortality	7 (2.9)	1 (0.3)	**0**.**008**
Preterm delivery	51 (23.9)	95 (29.7)	0.145
Apgar score <7	24 (9.8)	34 (9.9)	0.972
Small for gestational age	39 (15.9)	47 (13.7)	0.445
Foetal congenital heart disease	2 (0.8)	17 (4.9)	**0**.**005**
Neonatal mortality	5 (2)	5 (1.5)	0.587

Data are *n* (%), bold denotes *P* < 0.05. *P*-values were calculated between HDP and no HDP using χ^2^ tests. CS, Caesarean section; HDP, hypertensive disorders of pregnancy; HIC, high-income countries; LMIC, low-/middle-income countries.

## Discussion

### Principal findings and comparison to other studies

Prospective data from the ROPAC demonstrate that HDP occur more frequently than expected from the general population (10%^1,2^) in women with CMP, AOP, IHD, and PAH but not in women with CHD or VHD. The prevalence of pre-eclampsia was higher than expected (3–5% in the general population^1^) in women with CMP, IHD, and PAH. Women with heart disease and HDP had higher adverse maternal and perinatal outcomes than those without HDP. Even more strikingly, women with heart disease and pre-eclampsia had a maternal mortality rate of 3.5% and a heart failure rate of 29.1%. Thus, HDP affect women with heart disease differently and serious complications are reported, including maternal mortality (*Structured Graphical Abstract*).

### Prevalence of chronic hypertension and gestational hypertension

There is a large variation in the prevalence of chronic hypertension between different diagnostic groups; some heart conditions are directly associated with hypertension, such as aortic coarctation, others are indirectly linked through common cardiovascular risk factors, such as IHD.^3,4^ The prevalence of gestational hypertension that we observed does not seem higher than in the general population.^1^ It is notable that in this study, only 10.5% of women with chronic hypertension developed superimposed pre-eclampsia, as opposed to the 25% reported in the general population.^2^ A possible explanation is that they delivered (spontaneously or induced) earlier than the general population, as demonstrated by the 20.5% preterm births in women with chronic hypertension.

### Prevalence of pre-eclampsia

Pre-eclampsia is estimated to complicate 3–5% of pregnancies, whereas a global total range of 2–8% is described depending on the definition used and the population studied.^1^ While we found an overall prevalence of pre-eclampsia of 3%, there were differences between the various forms of structural heart disease. The prevalence seems similar to the background rate of the general population in women with VHD (2.2%)—except for aortic stenosis—and in CHD (2.6%)—except for pulmonary atresia. Our results are comparable with results from a meta-analysis, where pre-eclampsia affected 3.1% of pregnancies in women with CHD.^21^ Surprisingly, aortic coarctation, which is often complicated by chronic hypertension,^3^ did not increase the risk of pre-eclampsia. Although another study reported differences between complex and non-complex CHD (7.3 vs. 5.7%), the prevalence of pre-eclampsia was <5% even in complex CHD in our cohort, except for pulmonary atresia (15.6%).^13^

In comparison with the general population, the prevalence of pre-eclampsia was high among women with CMP (7.1%), PAH (11.1%), and IHD (6.3%). The population-based National Inpatient Sample in the United States also reported a high prevalence in CMP (25.6%) and PAH (22.3%), which is even more pronounced than our findings.^12^

We found surprisingly few differences in prevalence and outcomes between LMICs and HICs, given that pre-eclampsia is thought to be more prevalent in developing countries and a lack of access to care in LMICs is usually associated with adverse outcomes.^1^ There may be an underrepresentation of the prevalence and outcomes in LMICs because the centres that participate in the ROPAC may not be representative of rural prevalence and outcomes. Higher superimposed pre-eclampsia in LMICs could perhaps be due to less use of aspirin prophylaxis in women in chronic hypertension. Higher foetal CHD in HICs could be related to more advanced diagnostic modalities and follow-up during and after pregnancy.

### Pathogenesis of pre-eclampsia

Independent baseline predictors of pre-eclampsia in women with structural heart disease were overall comparable with the general population.^2,7^ In our cohort, we observed a relatively high proportion of early-onset, compared with late-onset, pre-eclampsia (1:3 vs. 1:7 in the general population),^22^ particularly in women with chronic hypertension, lower ejection fraction, and NYHA Class >II, but this may also be explained by our high rate of preterm births. The distinction between the two phenotypes is important, as they are thought to have different pathophysiological mechanisms: low cardiac output with a high vascular resistance and depleted intravascular fluids in early-onset pre-eclampsia, and high cardiac output with a normal-to-low vascular resistance and an intravascular fluid overload in late-onset pre-eclampsia; which also has consequences for the appropriate treatment.^23^

A higher prevalence of pre-eclampsia was associated with right-sided heart failure and venous congestion, as seen in pulmonary atresia (15.6%) and pulmonary hypertension (11.1%) but was not seen in women with a Fontan circulation (1.9%). In addition, some forms of left-sided heart disease such as aortic stenosis, CMP, and IHD had a higher prevalence of pre-eclampsia, whereas left ventricular function was not an independent predictor for pre-eclampsia. Moreover, not all women with known subclinical or clinical ventricular dysfunction developed pre-eclampsia.

Our findings partly support a pathophysiological role for cardiac dysfunction in the impairment of early pregnancy adaptations that leads to pre-eclampsia.^4,5,16^ However, our results also demonstrate that the relationship between cardiac function, placentation, placental perfusion, and pre-eclampsia development is not straightforward. Indeed, a complex interplay and other unknown factors likely explain why some women with ventricular dysfunction or venous congestion develop pre-eclampsia, whereas others do not.^24^ CMP and pre-eclampsia may share the pathophysiology of small vessel disease, including (pre-existing) endothelial damage, imbalanced angiogenic factors or a shared genetic predisposition, as certain protein altering genes that are present in both diseases.^25–27^ Oxidative stress could be another shared factor between cardiac disease and pre-eclampsia, because it is linked to vascular inflammation and endothelial dysfunction in cardiovascular disease,^28^ but it also plays an important role in the pathogenesis of pre-eclampsia.^4^

### Outcomes and clinical implications

Maternal and perinatal adverse outcomes were increased in women with pre-eclampsia. Maternal mortality occurred in 3.5%, which is much higher than expected and far above the maternal mortality in women with heart disease without pre-eclampsia (0.6%), and in pre-eclampsia in the general population (0.2–0.4%), both in HICs (0.1%) and LMICs (0.7%).^29–31^ Hypertensive disorders of pregnancy in the general population is associated with 1% severe cardiovascular morbidity, and as such, the observed incidence of complications is well beyond what would be expected for HDP.^32^ Women with heart disease and pre-eclampsia should be therefore followed by a multidisciplinary team with expertise in both the management of pre-eclampsia and of structural heart disease in pregnancy. Aspirin prophylaxis, which can reduce the risk of developing pre-eclampsia by up to 38% in the general population, seems justified in women with CMP, PAH, and IHD.^33^ A low threshold for the diagnostic workup for pre-eclampsia is indicated, as well as close clinical follow-up in the case of established pre-eclampsia. Adverse perinatal outcomes that we report herein, justify the systematic inclusion of a neonatologist in the multidisciplinary team, in preparation for the delivery. In the absence of a multidisciplinary heart team, long-distance digital or telephone consultations with a specialized centre would be warranted.

Since heart failure was the most common cause of death in our cohort, the cumulative risk of heart failure with structural heart disease and co-incident pre-eclampsia is likely to explain the observed high mortality rate. Indeed, the risk of developing heart failure is increased in both women with heart disease^11^ and in women with pre-eclampsia (OR: 11.9,^10^ owing to detrimental cardiac remodelling which causes both systolic and diastolic dysfunction,^34^ and an association with peripartum CMP and heart failure with preserved ejection fraction in particular).^10,35^ Our findings suggests that women with heart disease may have much less reserve to cope with the systemic endothelial dysfunction that occurs (or worsens, in case of pre-existing dysfunction) and increased afterload as a result of pre-eclampsia. Optimal heart failure therapy should promptly be instated and should not be delayed for concerns to the fetus.

In two cases of maternal death, there is a significant period of time between the diagnosis of pre-eclampsia and delivery. Although expectant management in early pre-eclampsia may be considered, a low threshold for delivery is necessary in case of clinical deterioration, irrespective of the gestational age. A risk model for adverse outcomes could help in clinical decision-making. In any case, delivery in women with heart disease is recommended at 40 weeks of gestation, which may also reduce the prevalence and associated risks of late pre-eclampsia in these women.^18^

Importantly, there is a need for continued vigilance even after delivery, as all deaths occurred in the post-partum period. In pre-eclampsia, the majority of maternal deaths occur in the first 6 weeks post-partum.^8^ Moreover, the incidence of heart failure among women with pre-eclampsia is at its highest in the immediate post-partum period because of the haemodynamic fluid shifts after delivery.^8,11^ Adequate and frequent post-partum follow-up is therefore crucial and should include attention to blood pressure, clinical signs of decompensation, and a re-evaluation of antihypertensive drugs and their dosage. We recommend clinical observation for at least 72 h and weekly or biweekly outpatient follow-up. Given their altered physiology, therapies developed to prevent or treat pre-eclampsia should be evaluated specifically in this complex subgroup of women with cardiac disease.

### Strengths and weaknesses of the study

Although the ROPAC cohort has a unique advantage in correctly classifying the types of HDP because—contrary to the general population—women with heart disease are usually already in medical care and their pre-pregnancy blood pressure is known, there are still several limitations that could cause a potential underrepresentation of HDP. Our data were collected by local investigators and there are likely regional differences in surveillance and ascertainment of HDP and other outcomes. To mitigate the risk of under-reporting, the most recent guidelines of the ISSHP were used to define HDP in the statistical analysis.^2^ The study design was not optimized to examine regional variations because the participation of countries among the geographic regions was variable and the numbers of pregnancies from some regions are low. In addition, there may exist a selection bias in the type of centre that participates in the ROPAC. Referral centres may treat more complex patients and therefore may have more adverse outcomes, which has a more pronounced effect when examining specific regions. The exact indication of cardiac medication use was not collected in the ROPAC, so we could not differentiate between drugs that were used in the treatment of the underlying cardiac disease and those that were used in the treatment of HDP. Antihypertensive drugs used for other indications (such as beta blockers for arrhythmia, AOP or heart failure) may have masked hypertension in some women, possibly causing an underrepresentation of chronic or gestational hypertension, and a misclassification of superimposed pre-eclampsia. The ROPAC data on aspirin use very likely substantially underestimate aspirin use on obstetric indication for the prevention of pre-eclampsia because it was listed in an unfolding menu that only appeared after indicating that the patient required anticoagulation. This makes the ROPAC data ill-suited to analyse the effect of aspirin in preventing pre-eclampsia or related complications. As HDP was not the primary outcome in the ROPAC study, HDP-specific clinical and laboratory parameters, and treatment modalities were not available. Contrary to the qualitative information on ventricular function, echocardiographic measurements were non-obligatory in the ROPAC and available in only 50% of pregnancies, and therefore could not be used in the analyses.

## Conclusions

The prospective and international ROPAC data show an increased risk of HDP in women with CMP, AOP, IHD, and PAH but not for women with CHD (except for pulmonary atresia) or VHD (except for aortic stenosis). This may be explained by a high prevalence of chronic hypertension in the high-risk groups. Moreover, the high prevalence of pre-eclampsia in CMP, IHD, and PAH may reflect shared risk factors and underlying predisposition. We thus recommend aspirin prophylaxis in these women, irrespective of the presence of chronic hypertension. Preventing pre-eclampsia in women with heart disease is of paramount importance, considering the increased risk of adverse maternal and perinatal outcomes, including a high mortality rate of 3.5%. Women should be counselled to recognize the signs and symptoms of pre-eclampsia and close attention and monitoring across the pregnancy continuum, including the post-partum period, would be warranted in a multidisciplinary context. More research is needed on how to prevent pre-eclampsia-related deaths in women with heart disease.

### One-sentence summary

The ROPAC data show high pre-eclampsia rates in women with pulmonary arterial hypertension (PAH), cardiomyopathy (CMP), and ischaemic heart disease (IHD), and adverse outcomes for all women with heart disease and HDP—in particular for pre-eclampsia, with 3.5% maternal mortality.

### Ethics approval

This study complies with the Declaration of Helsinki. Participating centres in the ROPAC managed the approvals of national or regional ethics committees or Institutional Review Boards, according to local regulations.

### Data sharing

The de-identified participant data that support the findings of this study are available from the corresponding author on reasonable request.

## Permissions information

The authors do hereby declare that all illustrations and figures in the manuscript are entirely original and do not require reprint permission.

## Supplementary Material

ehac308_Supplementary_Data
