# Supplementary material for: Hypertensive disorders of pregnant women with heart disease: the ESC EORP ROPAC Registry
Source: Eur Heart J. 2022 Jun 21;43(38):3749–61. doi: 10.1093/eurheartj/ehac308 (PMC9840477; doi:10.1093/eurheartj/ehac308)
Supplement: ehac308_Supplementary_Data [file ehac308_supplementary_data.pdf]

Supplementary file

Supplementary Table S1. Baseline pre-pregnancy characteristics of women with structural heart disease

	Total n=5739	CHD n=3295	VHD n=1649	CMP n=438	AOP n=217	IHD n=95	PAH n=45	*p*-value
Age, years (sd)	29.5 (5.6)	29 (5.4)	29.7 (5.8)	31 (5.8)	29.9 (5.3)	36.3 (5.7)	30.6 (5.4)	<0.001
BMI, kg/m^2 (Q1-Q3)	24 (21-28)	23 (21-27)	25 (22-29)	24 (22-28)	24 (21-27)	28 (25-34)	23 (21-28)	<0.001
Nulliparity	2573 (45)	1704 (52)	556 (34)	168 (38)	109 (51)	19 (20)	18 (40)	<0.001
Multiple pregnancy	96 (2)	46 (1.4)	33 (2)	10 (2)	4 (2)	1 (1)	2 (4)	0.309
LMIC	2281 (40)	936 (28)	1044 (63)	186 (43)	50 (23)	36 (38)	29 (64)	<0.001
Current smoker	228 (5)	132 (4)	38 (3)	29 (7)	8 (4)	18 (19)	1 (2)	<0.001
Chronic hypertension	380 (7)	183 (6)	75 (5)	55 (13)	27 (12)	30 (32)	10 (22)	<0.001
Diabetes mellitus	90 (2)	42 (1)	21 (1)	6 (1)	2 (1)	17 (18)	2 (4)	<0.001
Atrial fibrillation	106 (2)	16 (1)	86 (5)	3 (1)	1 (1)	0 (0)	0 (0)	<0.001
Signs of heart failure	596 (11)	213 (7)	273 (17)	80 (18)	5 (2)	11 (12)	14 (31)	<0.001
Estimated LVEF <40%	253 (4)	64 (2)	55 (3)	115 (26)	7 (3)	11 (12)	1 (2)	<0.001
NYHA class > II	204 (4)	61 (2)	98 (6)	36 (8)	0 (0)	3 (3)	6 (13)	<0.001
Cardiac medication use	2069 (36)	831 (25)	727 (44)	287 (66)	123 (57)	67 (71)	34 (76)	<0.001
Prior cardiac intervention	3160 (55)	2261 (69)	722 (44)	59 (14)	62 (29)	51 (54)	4 (9)	<0.001
mWHO I	1185 (21)	1055 (32)	130 (8)	0 (0)	0 (0)	0 (0)	0 (0)	<0.001
mWHO II	828 (14)	828 (25)	0 (0)	0 (0)	0 (0)	0 (0)	0 (0)	<0.001
mWHO II-III	2698 (47)	944 (29)	1070 (65)	382 (87)	208 (96)	94 (99)	0 (0)	<0.001
mWHO III	593 (10)	334 (10)	252 (15)	0 (0)	7 (3)	0 (0)	0 (0)	<0.001
mWHO IV	407 (7)	134 (4)	197 (12)	56 (13)	2 (1)	1 (1)	45 (100)	<0.001

Data are n (%), unless otherwise specified. *p*-values were calculated between the groups CHD, VHD, CMP, AOP, IHD and PAH using chi-square tests or one-way ANOVA as appropriate, significant if at least one of the groups is significantly different compared to the other groups. AOP, aortic pathology; BMI, Body Mass Index; CHD, congenital heart disease; CMP, cardiomyopathy; HDP, hypertensive disorders of pregnancy (defined as gestational hypertension and preeclampsia); IHD, ischemic heart disease; LMIC, low/middle-income country; LVEF, left ventricular ejection fraction; mWHO, modified World Health Organization classification for maternal cardiovascular risk; NYHA, New York Heart Association functional classification; PAH, pulmonary arterial hypertension; VHD, valvular heart disease.

Supplementary Table S2. Cardiac medication use in women with heart disease and HDP

	**ROPAC HDP n = 589**	
	Before pregnancy	During pregnancy
**Total**		
Beta blockers	111 (18.8)	155 (26.3)
ARBs	15 (2.5)	7 (1.2)
Diuretics	37 (6.3)	52 (8.8)
ACE-inhibitors	52 (8.8)	14 (2.4)
Antiarrhythmic drugs	5 (0.9)	11 (1.9)
Aldosterone blockers	9 (1.5)	7 (1.2)
Calcium channel blockers	32 (5.4)	56 (9.5)
Statins	13 (2.2)	5 (0.8)
Acetylsalicylic acid	40 (6.8)	64 (10.9)
Non-ASA antiplatelet drugs	8 (1.4)	10 (1.7)
Vitamin K antagonists	16 (2.7)	15 (2.5)
DOACs	1 (0.2)	2 (0.2)
Others	2 (0.4)	9 (1.5)

ARBs, angiontensin II blockers; ACE-inhibitor: angiotensin-converting-enzyme inhibitors; ASA, acetylsalicylic acid; DOAC, direct oral anticoagulants.

Supplementary Table S3. Main diagnosis of structural heart disease and the prevalence of superimposed preeclampsia and de novo preeclampsia.

	n	Superimposed preeclampsia	De novo preeclampsia	Total preeclampsia
**Total cohort**	**5739**	**40 (0.7)**	**132 (2.3)**	**172 (3)**
**CHD**	**3295**	**15 (0.5)**	**72 (2.2)**	**87 (2.6)**
Eisenmenger	31	0 (0)	1 (3.2)	1 (3.2)
Fontan circulation	54	0 (0)	1 (1.9)	1 (1.9)
Pulmonary atresia	32	0 (0)	5 (13.9)	5 (15.6)
Double outlet right ventricle	27	0 (0)	0 (0)	0 (0)
Tetralogy of Fallot	426	0 (0)	6 (1.4)	6 (1.4)
Congenitally corrected TGA	39	0 (0)	0 (0)	0 (0)
Atrial switch for TGA	121	0 (0)	4 (3.3)	4 (3.3)
Arterial switch for TGA	41	0 (0)	2 (4.9)	2 (4.9)
Aortic coarctation	303	6 (2)	2 (0.7)	8 (2.6)
Atrioventricular septal defect	169	0 (0)	3 (1.8)	3 (1.8)
Atrial septal defect	495	2 (0.4)	13 (2.6)	15 (3)
Ventricular septal defect	463	1 (0.2)	11 (2.5)	12 (2.6)
Ebstein’s anomaly	80	0 (0)	0 (0)	0 (0)
Aortic valve abnormality	267	2 (0.7)	9 (3.4)	11 (4.1)
Pulmonary valve abnormality	206	1 (0.5)	1 (1.9)	5 (2.4)
Mitral valve abnormality	83	0 (0)	0 (0)	0 (0)
Pulmonary vein abnormality	33	0 (0)	1 (3)	1 (3)
Patent ductus arteriosus	71	0 (0)	1 (1.4)	1 (1.4)
Other/unknown CHD	354	3 (1)	9 (2.2)	12 (3.4)
**VHD**	**1649**	**7 (0.4)**	**30 (1.8)**	**37 (2.2)**
Aortic stenosis	138	2 (1.4)	5 (3.6)	7 (5.1)
Aortic regurgitation	148	1 (0.7)	4 (2.7)	5 (3.4)
Mixed aortic disease	70	0 (0)	2 (2.9)	2 (2.9)
Mitral stenosis	288	0 (0)	3 (1)	3 (1)
Mitral regurgitation	500	2 (0.4)	5 (1)	7 (1.4)
Mixed mitral disease	278	0 (0)	7 (2.5)	7 (2.5)
Pulmonary stenosis	102	1 (1)	2 (2)	3 (2.9)
Pulmonary regurgitation	10	0 (0)	0 (0)	0 (0)
Other/unknown VHD	115	1 (0.9)	2 (1.7)	3 (2.6)
**CMP**	**438**	**10 (2.3)**	**21 (4.8)**	**31 (7.1)**
Dilated CMP	84	3 (3.6)	3 (3.6)	6 (7.1)
Hypertrophic CMP	93	0 (0)	1 (1.1)	1 (1.1)
Peripartum CMP	59	3 (5.1)	3 (5.1)	6 (10.2)
Myocarditis	19	1 (5.3)	0 (0)	1 (5.3)
Other/unknown CMP	183	3 (1.6)	14 (7.7)	17 (9.3)
**AOP**	**217**	**1 (0.5)**	**5 (2.3)**	**6 (2.8)**
Marfan syndrome	100	0 (0)	2 (2)	2 (2)

EDS type IV	4	0 (0)	0 (0)	0 (0)
Bicuspid aortic valve	44	1 (2.3)	0 (0)	1 (2.3)
Turner syndrome	16	0 (0)	0 (0)	0 (0)
FTAAD	3	0 (0)	0 (0)	0 (0)
Other/unknown AOP	50	0 (0)	3 (6)	3 (6)
**IHD**	**95**	**2 (2.1)**	**4 (4.2)**	**6 (6.3)**
**PAH**	**45**	**5 (11.1)**	**0 (0)**	**5 (11.1)**
Idiopathic/heritable	25	2 (8)	0 (0)	2 (8)
Connective tissue disease	3	0 (0)	0 (0)	0 (0)
Chronic thromboembolic PAH	4	0 (0)	0 (0)	0 (0)
Lung disease	2	0 (0)	0 (0)	0 (0)
Other/unknown	11	3 (27.3)	0 (0)	3 (27.3)
p*-value between diagnostic groups		<0.001	0.005	<0.001

Data are n (%). *p*-values were calculated using chi-square tests for the comparison between CHD, VHD, CMP, AOP, IHD and PAH, significant if at least one of the groups is significantly different compared to the other groups. HDP, hypertensive disorders of pregnancy (defined as gestational hypertension and preeclampsia); CHD, congenital heart disease; VHD, valvular heart disease; CMP, cardiomyopathy; IHD, ischemic heart disease; AOP, aortic pathology; PAH, pulmonary arterial hypertension; EDS, Ehlers-Danlos Syndrome; FTAAD, Familial Thoracic Aortic Aneurysm and Dissection; TGA, transposition of the great arteries.

Supplementary Table S4. Univariable and multivariable regression analysis of predictors of preeclampsia in women with structural heart disease.

	**OR**	**LL**	**UL**	**p-value**
**Univariable analysis**				
Age >35	1.8	1.3	2.6	**0.001**
BMI >30	1.6	1.0	2.5	**0.043**
Nulliparity	1.5	1.1	2.1	**0.006**
Multiple pregnancy	2.2	1.0	5.1	0.066
LMIC	1.1	0.8	1.5	0.565
Current smoker	1.5	0.8	2.8	0.270
Chronic hypertension	4.6	3.2	6.7	**<0.001**
Diabetes mellitus type 1/2	2.9	1.3	6.3	**0.009**
Signs of heart failure	1.7	1.1	2.5	**0.021**
Estimated LVEF <40	0.9	0.4	2.0	0.826
Pulmonary hypertension	1.9	1.2	2.8	**0.003**
NYHA class > II	2.3	1.3	4.1	**0.005**
Cardiac medication use	1.9	1.4	2.5	**<0.001**
HDP in previous pregnancy	3.7	2.0	6.9	**<0.001**
GDM in current pregnancy	3.0	1.7	5.4	**<0.001**
**Multivariable analysis**				
Age >35	1.6	1.1	2.3	**0.018**
BMI >30	1.3	0.8	2.2	0.248
Nulliparity	2.2	1.6	3.1	**<0.001**
Multiple pregnancy	2.3	1.0	5.4	0.063
Current smoker	1.2	0.6	2.5	0.544
Chronic hypertension	3.3	2.2	5.1	**<0.001**
Diabetes mellitus type 1/2	1.5	0.6	3.5	0.381
Signs of heart failure	1.2	0.7	1.9	0.534
Pulmonary hypertension	1.7	1.1	2.7	**0.021**
NYHA class > II	1.5	0.7	3.0	0.264
Cardiac medication use	1.3	0.9	1.8	0.149
HDP in previous pregnancy	2.4	1.2	4.8	**0.018**
GDM in current pregnancy	2.3	1.2	4.3	**0.009**

Bold denotes $p<0.05$. After multiple imputation for age (10.5% missing), BMI (56.6%), nulliparity (0.3%), smoking (16.5%), chronic hypertension (1.7%), diabetes mellitus (2.3%), signs of heart failure (1.5%), HDP in a previous pregnancy (0.6%), and gestational diabetes mellitus (1.2%). AOP, aortic pathology; BMI, Body Mass Index; CHD, congenital heart disease; CMP, cardiomyopathy; GDM, gestational diabetes mellitus; HDP, hypertensive disorders of pregnancy (defined as gestational hypertension and preeclampsia); IHD, ischemic heart disease; LL, lower limit of 95% confidence interval; LMIC, low/middle-income country; LVEF, left ventricular ejection fraction; mWHO, modified World Health Organization classification for maternal cardiovascular risk; NYHA, New York Heart Association functional classification; OR, odds ratio; PAH, pulmonary arterial hypertension; VHD, valvular heart disease; UL, upper limit of 95% confidence interval.

Supplementary Table S5. Comparison of maternal and perinatal outcomes between women with early and late preeclampsia

	**Early PE** n = 22	**Late PE** n = 64	**p-value**
**Maternal outcomes**			
Maternal mortality	0 (0)	1 (1.6)	0.555
Heart failure	8 (36.4)	17 (26.6)	0.382
Caesarean section	17 (85)	37 (57.8)	**0.027**
Emergency CS	10 (45.5)	22 (34.4)	0.354
Emergency CS for cardiac reasons	4 (18.2)	3 (4.7)	**0.046**
Postpartum haemorrhage	1 (4.5)	5 (7.8)	0.604
**Perinatal outcomes**			
Late fetal mortality	1 (4.5)	0 (0)	0.086
Preterm delivery	14 (70)	19 (29.7)	**0.001**
Apgar score <7	6 (27.3)	9 (14.1)	0.159
Small for gestational age	3 (13.6)	12 (18.8)	0.586
Fetal congenital heart disease	0 (0)	1 (1.6)	0.555
Neonatal mortality	1 (4.5)	1 (1.6)	0.423
Total perinatal mortality	2 (9.1)	1 (1.6)	0.097

Data are n (%), bold denotes $p<0.05$. *p*-values were calculated between early and late preeclampsia using chi-square tests. CS, Caesarean section; PE, preeclampsia.

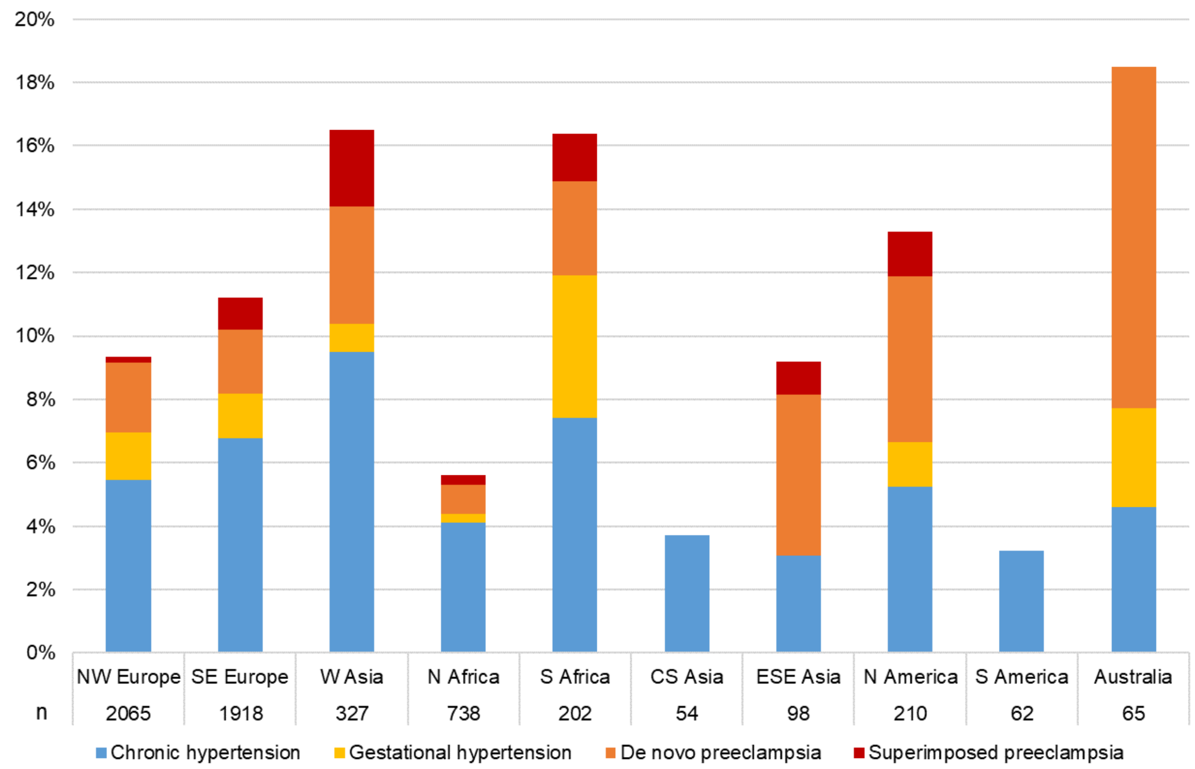

Supplementary Figure S1. Regional differences in the prevalence of HDP. p-values were calculated using chi-square tests for the comparison between the regions, <0.05 if at least one of the regions was significantly different compared to the other groups. NW Europe, Northern and Western Europe; SE Europe, Southern and Eastern Europe; W Asia, Western Asia; N Africa, Northern Africa; S, Southern Africa; CS Asia, Central Asia and Southern Asia; ESE Asia, Eastern Asia and Southeastern Asia; N America, Northern America; S America, Southern America.

	Northern & Western Europe	Southern & Eastern Europe	Western Asia	Northern Africa	Southern Africa	Central Asia & Southern Asia	Eastern Asia & Southeastern Asia	Northern America	Central & Southern America	Australia
	**n = 2065**	**n = 1918**	**n = 327**	**n = 738**	**n = 202**	**n = 54**	**n = 98**	**n = 210**	**n = 62**	**n = 65**
**Prevalence**										
Chronic hypertension	113 (5.5)	130 (6.8)	31 (9.5)	30 (4.1)	15 (7.4)	2 (3.7)	3 (3.1)	11 (5.2)	2 (3.2)	3 (4.6)
Gestational hypertension	31 (1.5)	27 (1.4)	3 (0.9)	2 (0.3)	9 (4.5)	0 (0)	0 (0)	3 (1.4)	0 (0)	2 (3.1)
Total preeclampsia	49 (2.4)	58 (3)	20 (6.1)	9 (1.2)	9 (4.5)	0 (0)	6 (6.1)	14 (6.7)	0 (0)	7 (10.8)
De novo preeclampsia	45 (2.2)	39 (2)	12 (3.7)	7 (0.9)	6 (3)	0 (0)	5 (5.1)	11 (5.2)	0 (0)	7 (1.8)
Superimposed preeclampsia	4 (0.2)	19 (1)	8 (2.4)	2 (0.3)	3 (1.5)	0 (0)	1 (1)	3 (1.4)	0 (0)	0 (0)
Total HDP	193 (9.3)	215 (11.2)	54 (16.5)	41 (5.6)	33 (16.3)	2 (3.7)	9 (9.2)	28 (13.3)	2 (3.2)	12 (18.5)
**Outcomes in women with HDP**	**n = 193**	**n = 215**	**n = 51**	**n = 41**	**n = 33**	**n = 2**	**n = 9**	**n = 28**	**n = 2**	**n = 12**
Maternal outcomes										
Maternal mortality	1 (0.5)	1 (0.5)	2 (3.7)	2 (4.9)	0 (0)	0 (0)	1 (11.1)	1 (3.6)	0 (0)	0 (0)
Heart failure	14 (7.3)	41 (19.1)	19 (35.2)	11 (26.8)	15 (45.5)	0 (0)	1 (11.1)	4 (14.3)	0 (0)	4 (33.3)
Caesarean section	102 (55.7)	136 (64.5)	36 (69.2)	25 (69.4)	19 (63.3)	2 (100)	7 (87.5)	11 (39.3)	1 (50)	6 (50)
Emergency CS	30 (15.5)	42 (19.5)	11 (20.4)	3 (7.3)	11 (33.3)	0 (0)	3 (33.3)	7 (25)	0 (0)	2 (16.7)
Emergency CS for cardiac reasons	4 (2.1)	9 (4.2)	4 (7.4)	0 (0)	3 (9.1)	0 (0)	0 (0)	0 (0)	0 (0)	1 (8.3)
Postpartum haemorrhage	6 (3.1)	4 (1.9)	5 (9.3)	1 (2.4)	0 (0)	0 (0)	0 (0)	0 (0)	1 (50)	1 (50)
Perinatal outcomes										
Late fetal mortality	1 (0.5)	0 (0)	3 (5.6)	3 (7.3)	1 (3)	0 (0)	0 (0)	0 (0)	0 (0)	0 (0)
Preterm delivery	43 (24.7)	54 (26.5)	19 (41.3)	5 (14.3)	8 (36.4)	0 (0)	3 (37.5)	12 (42.9)	0 (0)	2 (16.7)
Apgar score <7	14 (7.3)	23 (10.7)	2 (3.7)	3 (7.3)	5 (15.2)	0 (0)	3 (33.3)	6 (21.4)	0 (0)	2 (16.7
Small for gestational age	21 (10.9)	37 (17.2)	12 (22.2)	6 (14.6)	3 (9.1)	0 (0)	3 (33.3)	3 (10.7)	0 (0)	1 (8.3)
Fetal congenital heart disease	7 (3.6)	10 (4.7)	1 (1.9)	0 (0)	0 (0)	0 (0)	0 (0)	1 (3.6)	0 (0)	0 (0)
Neonatal mortality	3 (1.6)	3 (1.4)	2 (3.7)	1 (2.4)	0 (0)	0 (0)	0 (0)	1 (3.6)	0 (0)	0 (0)

Supplementary Table S6. Regional differences in the prevalence and outcomes of HDP

Data are n (%). p-values were calculated using chi-square tests for the comparison between the regions, <0.05 (bolded) if at least one of the regions was significantly different compared to the other groups. Countries were grouped into geographic regions as in the Report and Statistical Annex of the Sustainable Development Goals. CS, Caesarean section; HDP, hypertensive disorders of pregnancy

Appendix

ROPAC Executive Committee

Roger Hall GB (Co-Chair), Jolien Roos-Hesselink NL (Co-Chair), Joerg Stein, AT, William Anthony Parsonage, AU, Werner Budts, BE, Julie De Backer, BE, Jasmin Grewal, CA, Ariane Marelli, CA, Harald Kaemmerer, DE, Guillaume Jondeau, FR, Mark Johnson, GB, Aldo P. Maggioni, IT, Luigi Tavazzi, IT, Ulf Thilen, SE, Uri Elkayam, US, Catherine Otto, US, Karen Sliwa, ZA.

ROPAC Investigators

ARGENTINA - Buenos Aires: A. Aquieri, A. Saad, H. Ruda Vega, J. Hojman, J.M. Caparros, M. Vazquez Blanco **AUSTRALIA** - Elizabeth Vale: M. Arstall, C.M. Chung, G. Mahadavan, E. Aldridge, M. Wittwer, Y.Y. Chow, Herston: W.A. Parsonage, K. Lust, New Lambton Heights: N. Collins, G. Warner, R. Hatton, A. Gordon, E. Nyman **AUSTRIA** - Innsbruck: J. Stein , E. Donhauser , Vienna: H. Gabriel **AZERBAIJAN** - Baku: A. Bahshaliyev, F. Guliyev, I. Hasanova, T. Jahangirov, Z. Gasimov **BANGLADESH** - Dhaka: A. Salim, C.M. Ahmed, F. Begum, M.H. Hoque, M. Mahmood, M.N. Islam, P.P. Haque, S.K. Banerjee, T. Parveen **BELGIUM** - Brussels: M. Morissens, Gent: J. De Backer, L. Demulier, M. de Hosson, Leuven: W. Budts, M. Beckx **BOSNIA AND HERZEGOVINA** - Banja Luka: M. Kozic, M. Lovric, T. Kovacevic-Preradovic **BULGARIA** - Sofia: N. Chilingirova, P. Kratunkov **CANADA** - Edmonton: N. Wahab, S. McLean, Hamilton, Ontario: E. Gordon, L. Walter, Montreal: A. Marelli, A. R. Montesclaros **COLOMBIA** - Medellin: G. Monsalve, C. Rodriguez, F. Balthazar, V. Quintero, W. Palacio, L.A. Mejía Cadavid, E. Munoz Ortiz, F. Fortich Hoyos, E. Arevalo Guerrero, J. Gandara Ricardo, J. Velasquez Penagos **CZECH REPUBLIC** - Hradec Kralove: Z. Vavera, Prague: J. Popelova **DENMARK** - Copenhagen: N. Vejlstrup, L. Grønbeck, M. Johansen, A. Ersboll **EGYPT** - Alexandria: Y. Elrakshy, Assiut: K. Eltamawy , M. Gamal Abd-El Aziz, Benha : A. El Nagar, H. Ebaid, H. Abo Elenin, M. Saed, S. Farag, W. Makled, Cairo: K. Sorour, Z. Ashour, G. El-Sayed, M. Abdel Meguid Mahdy, Minia: N. Taha, A. Dardeer, M. Shabaan, Zagazig: A. Saad, M. Ali **FRANCE** - Nice: P. Moceri, Paris: G. Duthoit, M. Gouton, J. Nizard, L. Baris, S. Cohen, M. Ladouceur, D. Khimoud, B. Iung **GERMANY** - Berlin: F. Berger, A. Olsson, Bonn: U. Gembruch, W.M. Merz, E. Reinert, S. Clade, Y. Kliesch, Essen: C. Wald, Hamburg: C. Sinning, R. Kozlik-Feldmann, S. Blankenberg, E. Zengin-Sahm, G. Mueller, M. Hillebrand, P. Hauck, Y. von Kodolitsch, N. Zarniko, Muenster: H. Baumgartner, R. Schmidt, A. Hellige, Munich: O. Tutarel, H. Kaemmerer, B. Kuschel, N. Nagdyman, Oldenburg: R. Motz **GEORGIA** - Tbilisi: D. Maisuradze **GREECE** - Athens: A. Frogoudaki, E. Iliodromitis, M. Anastasiou-Nana, Marousi, D. Triantafyllis, G.

Bekiaris, Thessaloniki: H. Karvounis, G. Giannakoulas, D. Ntiloudi, S.A. Mouratoglou **HUNGARY** - Budapest: A. Temesvari, H. Balint, D. Kohalmi, B. Merkely, C. Liptai, Szeged: A. Nemes, T. Forster, A. Kalapos, K. Berek, K. Havasi, N. Ambrus **INDIA** - Karad: A. Shelke, R. Kawade, S. Patil **INDONESIA** - Bandung: E. Martanto, T.M. Aprami, A. Purnomowati, C.J. Cool, M. Hasan, R. Akbar, S. Hidayat, T.I. Dewi, W. Permadi, D.A. Soedarsono **IRAN** - Tehran: M.M. Ansari-Ramandi, N. Samiei, A. Tabib, F. Kashfi, S. Ansari-Ramandi, S. Rezaei **IRAQ** - Baghdad: H. Ali Farhan, A. Al-Hussein, G. Al-Saedi, G. Mahmood, I.F. Yaseen, L. Al-Yousuf, M. AlBayati, S. Mahmood, S. Raheem, T. AlHaidari, Z. Dakhil **IRELAND** - Dublin: P. Thornton, J. Donnelly, M. Bowen **ISRAEL** - Beer Yakov: A. Blatt, G. Elbaz-Greener, Hadera: A. Shotan, Haifa: S. Yalonetsky, Rehovot: S. Goland, M. Biener **ITALY** - Bologna: G. Egidy Assenza, M. Bonvicini, A. Donti, A. Bulgarelli, D. Prandstraller, Bolzano: C. Romeo, R. Crepaz, Brescia: E. Sciatti, M. Metra, R. Orabona, Massa: L. Ait Ali, P. Festa, Milan: V. Fesslova , C. Bonanomi, M. Calcagnino, F. Lombardi, A.M. Colli, M.W. Ossola, C. Gobbi, E. Gherbesi, L. Tondi, M. Schiavone, M. Squillace, Palermo: M.G. Carmina, Torino: A. Maina, C. Macchi, E. Gollo, F.M. Comoglio, N. Montali, P. Re, R. Bordese, T. Todros, V. Donvito, W. Grosso Marra, Trieste: G. Sinagra, B. D'Agata Mottolese, M. Bobbo, V. Gesuete, S. Rakar, F. Ramani **JAPAN** - Chiba: K. Niwa **KAZAKHSTAN** - Almaty: D. Mekebekova, A. Mussagaliyeva, T. Lee **KYRGYZSTAN** - Bishkek: E. Mirrakhimov, S. Abilova, E. Bektasheva, K. Neronova, O. Lunegova **LITHUANIA** - Kaunas: R. Žaliūnas, R. Jonkaitienė, J. Petrauskaitė, Vilnius: A. Laucevicius, D. Jancauskaite, L. Lauciuviene, L. Gumbiene, L. Lankutiene, S. Glaveckaite, M. Laukyte, S. Solovjova, V. Rudiene **MALAYSIA** - Kuala Lumpur: K.H. Chee, C.C-W. Yim, H.L. Ang, R. Kuppusamy, T. Watson **MALTA** - Birkirkara: M. Caruana **NORWAY** - Oslo: M-E. Estensen **PAKISTAN** - Rawalpindi: M.G.A. Mahmood Kayani, R. Munir **POLAND** - Bialystok: A. Tomaszuk-Kazberuk, B. Sobkowicz, J. Przepiesc, Krakow: A. Lesniak-Sobelga, L. Tomkiewicz-Pajak, M. Komar, M. Olszowska, P. Podolec, S. Wisniowska-Smialek, Lodz: M. Lelonek, U. Faflik, A. Cichocka-Radwan, Poznan: K. Plaskota, O. Trojnarska **PORTUGAL** - Coimbra: N. Guerra, Lisboa: L. de Sousa, Porto: C. Cruz, V. Ribeiro **REPUBLIC OF MACEDONIA** - Skopje: S. Jovanova **ROMANIA** - Bucharest: V. Petrescu, R. Jurcut, C. Ginghina, I. Mircea Coman, M. Musteata **RUSSIA** - Belgorod: O. Osipova, T. Golivets, I. Khamnagadaev, O. Golovchenko, A. Nagibina, I. Ropatko, Izhevsk: I.R. Gaisin, L. Valeryevna Shilina, Moscow: N. Sharashkina, Saint- Petersburg: E. Shlyakhto, O. Irtyuga, O. Moiseeva, E. Karelkina, I. Zazerskaya, A. Kozlenok, I. Sukhova **SERBIA** - Belgrade: L. Jovovic **SLOVENIA** - Ljubljana: K. Prokšelj, M. Koželj **SOMALILAND** - Hargeisa: A.O. Askar, A.A. Abdilaahi, M.H. Mohamed, A.M. Dirir **SOUTH AFRICA** - Cape Town: K. Sliwa, Houghton: P. Manga **SPAIN** - Barcelona: A. Pijuan-Domenech, L.

Galian-Gay, P. Tornos, M.T. Subirana, M. T. Subirana , Bilbao: N. Murga, Madrid: J. M. Oliver, B. Garcia-Aranda Dominguez, I. Hernandez Gonzalez, J.F. Delgado Jimenez, P. Escribano Subias **SUDAN** - Khartoum: A. Elbushi, A. Suliman, K. Jazzar, M. Murtada, N. Ahamed **SWEDEN** - Göteborg: M. Dellborg, E. Furenas, M. Jinesjo, K. Skoglund, P. Eriksson, T. Gilljam, Lund: U. Thilen **SWITZERLAND** - Basel: D. Tobler, Bern: K. Wustmann, F. Schwitz, M. Schwerzmann, Lausanne: T. Rutz, J. Bouchardy, Zurich: M. Greutmann, B.M. Santos Lopes, L. Meier, M. Arrigo **THE NETHERLANDS** - Amsterdam: K. de Boer, T. Konings, Enschede: E. Wajon, L.J. Wagenaar, Geldrop: P. Polak, Groningen: E.PG. Pieper, Rotterdam: J. Roos-Hesselink, L. Baris, I. van Hagen, H. Duvekot, J.M.J. Cornette, The Hague: C. De Groot, Utrecht: C. van Oppen **TURKEY** - Istanbul: L. Sarac, O. Batukan Esen, S. Catirli Enar **UGANDA** - Kampala: C. Mondo, P. Ingabire, B. Nalwanga, T. Semu **UNITED ARAB EMIRATES** - Abu Dhabi: B.T. Salih, W.A.R. Almahmeed, S. Wani, F.S. Mohamed Farook, Al Ain, F. Gerges, A.M. Komaranchath, F. Al bakshi, Dubai: A. Al Mulla, A.H. Yusufali, E.I. Al Hatou, N. Bazargani, F. Hussain **UNITED KINGDOM** - Birmingham: L. Hudsmith, P. Thompson, S. Thorne, S. Bowater, Buckinghamshire: A. Money-Kyrle, P. Clifford, P. Ramrakha, S. Firoozan, J. Chaplin, N. Bowers, Coventry: D. Adamson, London: F. Schroeder, R. Wendler, S. Hammond, P. Nihoyannopoulos, Norwich Norfolk: R. Hall, L. Freeman, Southampton: G. Veldtman, J. Kerr, L. Tellett **UNITED STATES** - Boston: N. Scott, A.B. Bhatt, D. DeFaria Yeh, M.A. Youniss, M. Wood, A.A. Sarma, S. Tsiaras, A. Stefanescu, J.M. Duran, L. Stone, Cleveland: D.S. Majdalany, J. Chapa, Detroit: K. Chintala, P. Gupta , Hershey, PA: J. Botti, J. Ting, W. R. Davidson, Lexington, Kentucky: G. Wells, D. Sparks, Mineola, NY: V. Paruchuri, K. Marzo, D. Patel, Minneapolis: W. Wagner, S.N. Ahanya, L. Colicchia, T. Jentink, K. Han, M. Loichinger, M. Parker, W. Wagner, C. Longtin, Omaha: A. Yetman, K. Erickson, J. Cramer, S. Tsai, B. Fletcher, S. Warta, Phoenix: C. Cohen, C. Lindblade, R. Puntel, K. Nagaran, N. Croft, Seattle: M. Gurvitz, C. Otto, Stanford, CA: C. Talluto, D. Murphy, M. G. Perlroth.
